# Exploring the Survival Determinants in Recurrent Ovarian Cancer: The Role of Cytoreductive Surgery and Hyperthermic Intraperitoneal Chemotherapy

**DOI:** 10.3390/cancers16112150

**Published:** 2024-06-05

**Authors:** Katarzyna Gęca, Jakub Litwiński, Tomasz Ostrowski, Izabela Świetlicka, Wojciech P. Polkowski, Magdalena Skórzewska

**Affiliations:** 1 Department of Surgical Oncology, Medical University of Lublin, Radziwiłłowska 13 St., 20-080 Lublin, Poland; klitwinski@gmail.com (J.L.);; 2 Department of Biophysics, University of Life Sciences, Akademicka 13, 20-950 Lublin, Poland; izabela.swietlicka@up.lublin.pl

**Keywords:** recurrent ovarian cancer, cytoreductive surgery, hyperthermic intraperitoneal chemotherapy, HIPEC, AGO score

## Abstract

**Simple Summary:**

Ovarian cancer ranks as the second most prevalent genital malignancy in women and leads to higher rates of mortality among gynecological cancers, often diagnosed at advanced stages with extensive metastases. Historically, treatment for recurrent ovarian cancer (ROC) has focused on managing peritoneal metastases, typically through aggressive surgery followed by systemic chemotherapy. Recent advancements have included secondary cytoreductive surgery and the use of hyperthermic intraperitoneal chemotherapy (HIPEC), aimed at improving survival but still debated due to variable outcomes in prior studies. This study conclusively finds that factors such as radical surgery, good performance status, platinum sensitivity, a positive AGO score, and a low Peritoneal Carcinomatosis Index (PCI) significantly enhance survival rates for patients with ROC undergoing HIPEC. It also highlights the predictive importance of platinum resistance and the AGO score in determining outcomes. Given these results, there is a strong recommendation for further prospective studies to validate these findings and to refine the criteria for selecting patients suitable for HIPEC, aiming to improve the treatment strategies and survival outcomes in this patient group.

**Abstract:**

Background: Recurrent ovarian cancer (ROC) significantly challenges gynecological oncology due to its poor outcomes. This study assesses the impact of cytoreductive surgery (CRS) combined with hyperthermic intraperitoneal chemotherapy (HIPEC) on ROC survival rates. Materials and Methods: Conducted at the Medical University of Lublin from April 2011 to November 2022, this retrospective observational study involved 71 patients with histologically confirmed ROC who underwent CRS and subsequent HIPEC. Results: The median overall survival (OS) was 41.1 months, with 3-year and 5-year survival rates post-treatment of 0.50 and 0.33, respectively. Patients undergoing radical surgery for primary ovarian cancer had a median OS of 61.9 months. The key survival-related factors included the Peritoneal Carcinomatosis Index (PCI) score, AGO score, platinum sensitivity, and ECOG status. Conclusions: The key factors enhancing ROC patients’ survival include radical surgery, optimal performance status, platinum sensitivity, a positive AGO score, and a lower PCI. This study highlights the predictive value of the platinum resistance and AGO score in patient outcomes, underlining their role in treatment planning. Further prospective research is needed to confirm these results and improve patient selection for this treatment approach.

## 1. Introduction

Ovarian cancer (OC) is the second most common genital malignancy in females and accounts for the majority of deaths from gynecologic malignancies. To date, no screening methods to detect OC at an early stage have been developed; thus, approximately 60–70% of women with newly diagnosed OC present with advanced stage disease—FIGO III and IV—characterized by extensive peritoneal spread or distant metastases [1,2]. The prognosis for OC remains daunting, with a mere 17% of patients at an advanced stage surviving beyond five years [3,4].

In recent years, there has been a heightened focus on the examination of treatment protocols for peritoneal metastases (PMs) in recurrent ovarian cancer (ROC), which are closely linked to the presence of peritoneal cancer implants. The primary management of OC involves macroscopic complete resection as the fundamental approach, followed by adjuvant platinum/taxane-based systemic chemotherapy (CTH), primarily with carboplatin and paclitaxel [5]. The prognostic significance of surgical thoroughness cannot be overstated, critically influencing survival [6]. Nevertheless, despite meticulous surgical cytoreduction combined with CTH, a relapse occurs in over half of all patients diagnosed at FIGO stage III. The evidence suggests that ROC substantially impacts survival rates, particularly in individuals affected by PMs [7].

The role of secondary cytoreductive surgery in ROC has been under debate for decades. Prior to the DESKTOP series of trials, the available data, predominantly retrospective, catered to a heterogeneous patient cohort. DESKTOP-I illuminated the prognostic benefit conferred by macroscopic complete resection in ROC cases [8]. Consequently, the Arbeitsgemeinschaft Gynäkologische Onkologie (AGO) score was developed, incorporating three prognostic variables for complete resection (ECOG performance status of 0; ascites below 500 mL; and complete resection in primary surgery) [8]. The prognostic score was validated by the DESKTOP-II trial, while the DESKTOP-III trial emphasized the superiority of combining cytoreductive surgery (CRS) with systemic CTH over CTH alone in ROC. The achievement of macroscopic complete resection resulted in a median overall survival (OS) of 61.9 months for 75.5% of the participants [5,9]. These findings underscore the crucial importance of top-tier surgical intervention in maximizing patient outcomes in ROC.

While hyperthermic intraperitoneal chemotherapy (HIPEC) combined with interval cytoreductive CRS and neoadjuvant CTH shows notable improvements in OS and disease-free survival (DFS) in primary OC, its benefits in recurrent settings are less clear, showing no significant survival advantage [10]. Despite promising results in primary settings, the use of HIPEC for ROC remains controversial, with ongoing trials like OVHIPEC-2 expected to provide more definitive answers [11,12]. The recently updated ESMO-ESGO-ESP consensus recommends not to perform HIPEC in cytoreductive surgery for relapsed disease [13]. Patient selection and procedure specifics are crucial for HIPEC’s effectiveness, necessitating further research for standardization [14]. Current data support the use of HIPEC only after optimal interval cytoreductive surgery in advanced stages, emphasizing the need for strict adherence to evidence-based guidelines and more research to refine these recommendations [15].

The application of HIPEC in ROC remains a nuanced and debated topic within the scientific community. The current guidelines suggest HIPEC as an option for experimental studies. This study aims to evaluate the long-term therapeutic outcomes in patients with ROC who underwent CRS combined with HIPEC.

## 2. Materials and Methods

This retrospective, observational study was conducted after obtaining institutional review board approval (Bioethical Committee of the Medical University of Lublin, Ethic Code: KE—0254/331/2018). Written informed consent was obtained from the patients following the principles outlined in the Declaration of Helsinki. This study was designed and reported in accordance with the Strengthening the Reporting of Observational Studies in Epidemiology (STROBE) guidelines to ensure clarity, transparency, and reproducibility. The records were retrieved from the database of patients with ROC treated between April 2011 and November 2022 at the Department of Surgical Oncology, Medical University of Lublin, Poland.

**Figure 1:**
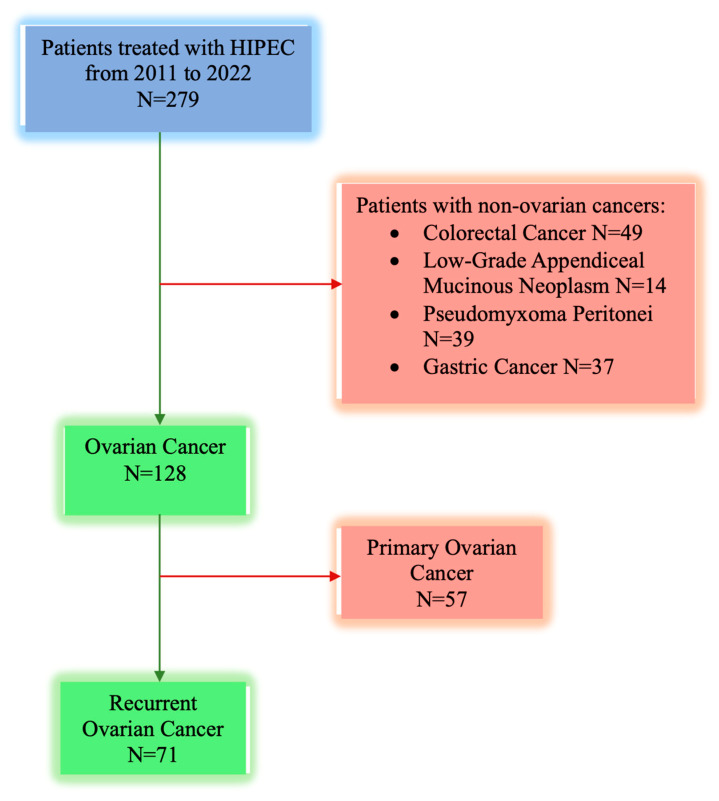
Flow chart representing the patient selection process.

The multidisciplinary team board meetings addressed the management of all the patients. The final analysis included a cohort of seventy-one eligible patients. The patient selection process is presented in Figure 1.

Inclusion Criteria:

- Age ≥ 18 years
- Histologically confirmed diagnosis of the first recurrence of epithelial OC
- Status post-surgery for primary OC with adjuvant platinum-based CTH
- Recurrence confirmed via radiology with/without elevated CA-125 and HE4 levels
- Al least one measurable target lesion per RECIST v 1.1
- ECOG performance score of 0–2, indicating ability for self-care and ambulatory for over 50% of waking hours
- Adequate organ and bone marrow function for CRS and HIPEC treatment
- Deemed suitable for CRS and HIPEC by a multidisciplinary team
- Written informed consent for treatment

Exclusion Criteria:

- Diagnosis of non-epithelial OC like germ cell or stromal tumors
- Stage IV disease marked by extra-abdominal metastasis
- Previous treatment with CRS and HIPEC for OC
- Serious cardiac, lung, kidney, or liver conditions posing surgery risks
- Other active primary cancers
- ECOG status of 3 or higher, indicating severe incapacitation
- Underlying medical or psychiatric conditions that will make the administration of therapy hazardous
- Dementia, psychiatric, or substance abuse disorders that would interfere with treatment
- Known untreated, symptomatic, or actively progressing central nervous system metastases
- Known acute hepatitis B, known chronic hepatitis B infection with active untreated disease, or known hepatitis C infection
- Life expectancy under 6 months

### 2.1. Platinum Sensitivity

Platinum-sensitive disease is characterized by a recurrence that occurs more than 6 months after the administration of standard first-line CTH [16]. Patients with platinum-sensitive recurrence were treated with platinum-based CTH. Paclitaxel, topotecan, gemcitabine, etoposide, or liposomal doxorubicin were administered in cases of platinum-resistant recurrences.

### 2.2. The Peritoneal Carcinomatosis Index and Cytoreduction Completeness

The Peritoneal Carcinomatosis Index (PCI) was utilized for the purpose of staging [17]. The score, ranging from 0 to 39 points, represents the size of the tumor lesions across 13 areas within the peritoneal cavity. Cytoreduction completeness (CC) was assessed using a four-point scale: CC0 denotes complete cytoreduction, CC1 signifies residual tumor nodules measuring less than 2.5 mm, CC2 refers to residual tumor nodules ranging from 2.5 mm to 2.5 cm, and CC3 indicates remaining lesions larger than 2.5 cm. The effective use of HIPEC is contingent upon CC0 or CC1 resections, as the potent cytotoxic effect can only be anticipated in cases where there is no visible tumor (CC0) or minimal residual tumor nodules (not exceeding 2.5 mm). Peritonectomy was employed to carry out cytoreductive surgery in every anatomical region of the abdomen and/or pelvis where a PM is present. The extent of resection exhibited significant variability due to the quantity and locations of the PMs. It involved the performance of the following procedures: partial or total peritonectomy (pelvic and/or diaphragmatic), greater or minor omentectomy, right- or left-sided hemicolectomy, anterior resection of the rectum, splenectomy, partial excision of the small intestine, and excision of solitary peritoneal metastases.

### 2.3. HIPEC Procedure

Intraperitoneal perfusions either with open “Coliseum” or close techniques were performed [18]. HIPEC was administered at a rate of 4–11 L per minute, maintaining a temperature of 42–43 °C, utilizing the SunChip^®^ 1.0 system (Gamidatech, Eaubonne, France). Two cytostatic drugs were used for the intraperitoneal perfusion: Mitomycin C at a dose of 30 mg with the 0.9% NaCl solution as the solvent for 60 min or Oxaliplatin at a dose of 300 mg/m^2^ with the 5% glucose as the solvent, for 30 or 45 min.

### 2.4. AGO Score

The AGO score was utilized to retrospectively assess the prognostic factors and outcomes of patients with ROC. A positive AGO score, which indicates a favorable prognosis for surgery, is achieved if a patient meets all of the following three conditions: complete resection at first surgery (or FIGO stage I/II), good performance status (ECOG performance status of 0 or 1), and absence of ascites greater than 500 mL. Conversely, a negative AGO score indicates that one or more of these conditions are unmet, suggesting a less favorable prognosis for surgery [5,8,9].

### 2.5. Statistical Analysis

The collected data were analysed concerning their nature. Categorical variables were characterised in terms of the structure indices, namely the absolute counts and percentage shares (%). The normal distribution (Shapiro–Wilk test) and variance homogeneity (Levene’s test) were checked for the continuous variables that, due to the skewness, were presented as means ± standard error (SE) with confidence intervals (C.I.), medians, and upper (Q1) and lower (Q3) quartiles. Continuous variables were categorized when needed.

The survival times were compared across the groups according to a range of variables using Kaplan–Meier analysis, which estimates the survival probability over time. The survival probabilities, median times of survival and quartile estimates were determined for all the variables and compared using the log-rank test and pairwise comparison (at *p* < 0.05). Patients still alive or those who were lost to follow-up at the end of the study period were right-censored. The survival time was defined as the time from the HIPEC treatment until either death or the time of the last follow-up. The null hypothesis that the distribution curves do not differ at all time points among the groups was tested against the alternative hypothesis, which states that all the groups have different distribution curves. This approach allows for the assessment of differences in the survival distributions across the groups, helping to identify statistically significant variations in survival times between different categories or treatment groups. Additionally, the effect sizes (ES) were calculated according to Equation (1), which was proposed in the study conducted by Wang et al. [19]. The ES is equal to the probability that a randomly selected subject from Group 2 can be observed to live longer than a randomly selected subject from Group 1 minus the probability that a randomly selected subject from Group 1 can be observed to live longer than a randomly selected subject from Group 2 and takes a value between −1 and 1 (a negative/positive effect size ESG implies a lower/higher hazard in Group 1 than in Group 2). What is more, it is dependent on the survival functions, capturing the dynamic changes in the survival probability over time.

(1) $$ES=\frac{n}{n_{1}n_{2}}\sum_{j=1}^{J}\frac{Y_{j}}{n}\left(\frac{D_{1j}Y_{2j}}{Y_{j}}-\frac{D_{2j}Y_{1j}}{Y_{j}}\right)$$

where n_1_—the number of subjects in sample 1, n_2_—the number of subjects in sample 2, n—the total number of subjects, D_1j_—the number of subjects who failed at tj in sample 1, D_2j_—the number of subjects who failed at tj in sample 2, Y_1j_—the number of subjects who were at risk at tj in sample 1, Y_2j_—the number of subjects who were at risk at tj in sample 2, and Y_j_—the total number of subjects who were at risk at tj in both samples. This calculation allows for the quantification of the magnitude of the difference in survival times between the groups, providing a more precise understanding of the comparative hazards faced by each group.

The estimated survival probabilities at the fixed time points t_01_ = 3 years (36 months) and t_02_ = 5 years (60 months) were then compared using the estimated survival probabilities after adjusting for other covariates using the method presented by Zhang et al. and Klein et al. [20]. When there were more than two groups, similar survival probabilities were combined at a given time point.

Univariate and multivariable Cox proportional hazard (PH) models were used to investigate the prognostic factors, which allowed for identifying the individual and combined impact of various predictors on the survival time. The existence of interactions between the variables and their effects over time was examined, providing insights into how these variables might influence each other and the OS outcomes. The Cox PH assumption, which requires that the hazard ratios between groups remain constant over time, was assessed using the scaled Schoenfeld residuals to ensure the validity of the model. Influential observations were checked with the dfBETA, which measures the change in the estimated regression coefficients when the model is fitted using all the observations versus when we fit the model leaving out one observation at a time according to Equation (2):

(2) $$dfBETA\;A_{ij}=\hat{\beta_{J}}-\hat{\beta_{\mathrm{IJ}}}$$

where $\hat{\beta_{J}}$ –the jth coefficient from the regression calculated using all the data, and $\hat{\beta_{IJ}}$—the jth coefficient from the regression calculated without i^th^ observation. The dfBETA threshold was set at $\frac{2}{\sqrt{n}}\cdot SE$, where n is the number of observations and SE is the standard error. This attitude helps in detecting any data points that disproportionately affect the model’s estimates.

Finally, principal component analysis (PCA) was applied to identify prognostic factors as a combination of variables that are most significant for predicting the outcome and help to stratify patients into different risk groups based on their scores for the first few principal components. This technique reduces the dimensionality of the data by transforming correlated variables into a smaller number of uncorrelated components, thus simplifying the analysis while retaining most of the variability in the data. The PCA model was built based on cases defined as uncensored, while the censored were projected into a new space as additional data, ensuring that all the available information was utilized effectively. The validity of the factor analysis was assessed using the Bartlett statistics and Kaiser–Meyer–Olkin (KMO) test. Bartlett’s test checks whether the correlation matrix is significantly different from an identity matrix, indicating that the variables are sufficiently correlated for PCA. The KMO test measures the adequacy of the sampling, with values closer to 1 indicating that PCA is appropriate. The input data matrix was scaled to the unitary standard deviation, meaning that PCA was performed on the correlation matrix and all the variables were treated equally. The principal components were determined using v-fold cross-validation, a method that enhances the reliability of the component selection process by repeatedly partitioning the data into subsets and validating the model on each. The scree plots, which graphically display the eigenvalues associated with each principal component, the total variance explained, and the Kaiser–Guttman criterion were used to select the optimum number of components.

All the statistical evaluations were performed using a two-sided approach, with a significance threshold of α = 0.05. The statistical assessments were conducted using RStudio (2023.09.1, build 494, Integrated Development for R. RStudio, PBC, Boston, MA, USA URL http://www.rstudio.com/.), STATISTICA 13.0 (Dell Technologies Inc., Round Rock, TX, USA), and OriginPro 2022 (OriginLab, MA, USA) software.

## 3. Results

### 3.1. Study Group Characteristic

The cohort, whose characteristics are showcased in Table 1 and Table 2, comprising 71 female patients diagnosed with ROC, exhibited a mean survival time of 41.1 months (Table 1), with a standard error of ±3.8 months (median—31 months). The primary surgery was performed on all the patients with varying levels of radicality. In the studied cohort (Table 2), 31% of the patients (numbering 22) underwent non-radical surgery for primary OC (N), whereas the remaining 69%, comprising 49 patients, were subjected to radical surgical procedures (R). Platinum resistance was not observed in over 80% of the patients. According to the ECOG performance status scale (Table 2), 76% of patients were assessed with a score of “0”, indicating full functionality; 17% with a score of “1”, indicative of slight functional impairment; and 7% (5 patients) with a score of “2”, denoting a greater level of functional compromise. Given the small number of patients with scores of “1” or “2”, these groups were combined for further analysis. An AGO score (Table 2) assessment rendered 42 patients (59%) positive scores, whereas the remaining 29 (41%) were ascribed negative ones. The completeness of the CCR index was predominantly scored as CC0 in 80% of patients, followed by 15.5% allocated to the CC1 category. Two individuals were classified as CC2 and a solitary patient was categorized as CC3. Patients with CCR indices ranging from 1 to 3 were considered a single group for analytical uniformity.

Two chemotherapeutic agents (Table 2) were utilized in the HIPEC procedure, with Mitomycin C employed in 42% of cases and Oxaliplatin used in the remaining cases. The mean CRS time was equal to 159.86 (±6.63) minutes, while the HIPEC procedure took an average of 39.30 (±1.45) minutes (Table 1). The administration technique was dichotomized (Table 2) into open (54%) and closed (46%).

### 3.2. Kaplan–Meier Analysis

**Figure 2:**
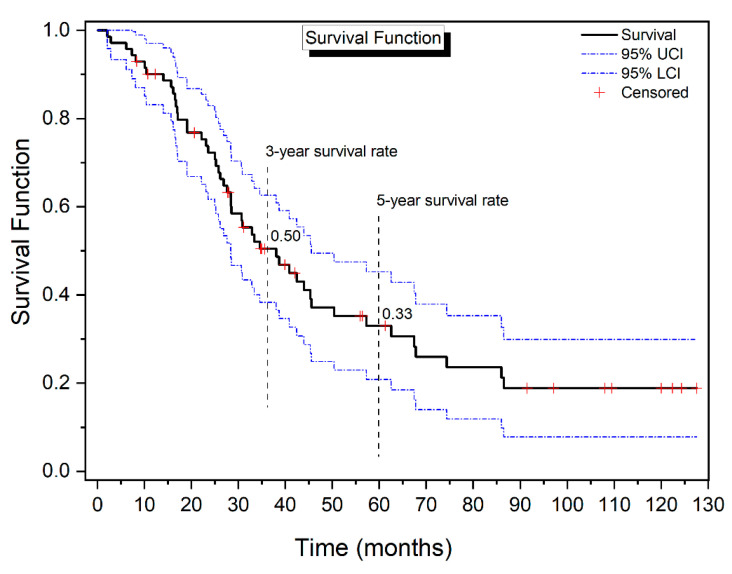
Overall survival function (black line) with 95% confidence intervals (blue, dotted line) for women diagnosed with ovarian cancer after HIPEC therapy. Observations assigned a + were right-censored.

The Kaplan–Meier survival analysis yielded an estimated mean survival duration of 52.65 ± 5.42 months for the entire patient cohort following the applied treatment, with a 95% confidence interval ranging from 42.03 to 63.28 months. The quartile estimations indicate the fact that 25% of patients had succumbed by the 23-month mark, while the median time to death for 50% of the cohort was observed at 38 months, and 75% had reached this event by 74 months. The analysis also delineated the survival probabilities at specified time points, revealing 3-year and 5-year survival rates at 0.50 and 0.33, respectively (Figure 2, Table 3).

**Figure 3:**
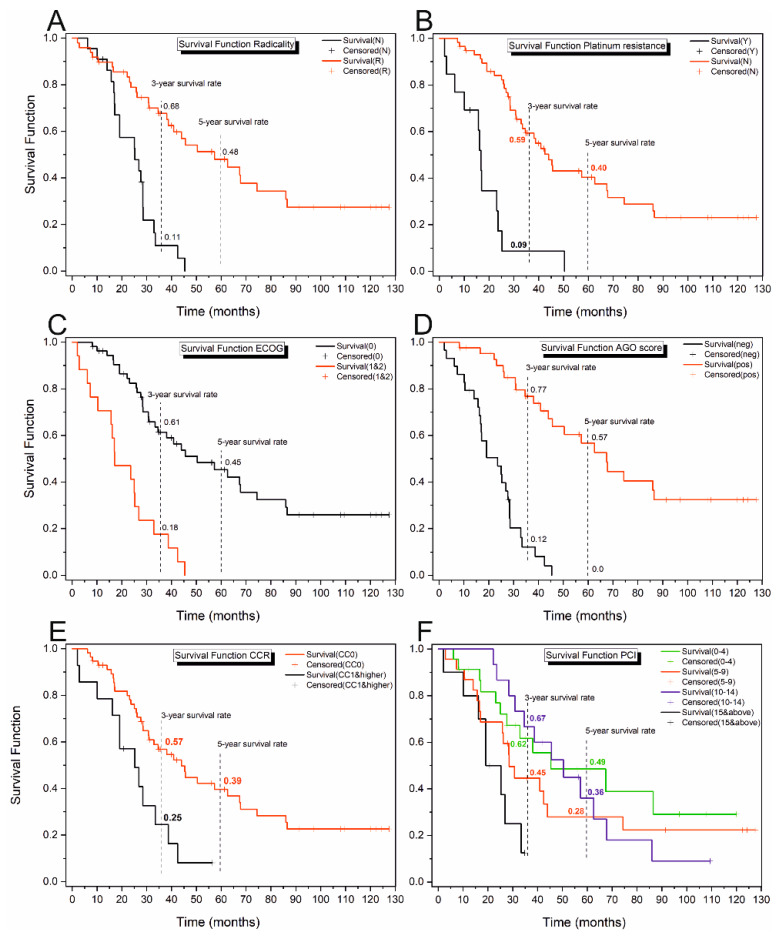
Survival curves with 3-year and 5-year survival rates determined for the patient cohort: (**A**) subjected to the radical (R, red line) or non-radical (N, black line) primary surgery, (**B**) platinum sensitive (N, red line) and resistant (Y, black line), (**C**) according to ECOG performance status scale (0—the black line, 1 and 2—the red line), (**D**) according to AGO score (negative—the black line, positive—the red line); (**E**) according to the CCR index (CC0—the red line, CC1 and higher—the black line) and (**F**) regarding PCI (0–4 the green line, 5–9 the red line, 10–14 the blue line, 15 and above the black line). Observations assigned a + were right-censored.

Survival in patients with ROC was examined concerning the radicality of the primary surgery (N/R), platinum resistance (Y/N), ECOG (0, 1–2), AGO score (positive/negative), CCR (0, 1–3), CRS time, type of cytostatic (Mitomycin C and Oxaliplatin) and method of HIPEC procedure (open/closed), HIPEC time and PCI. The investigation results produced using Kaplan–Meier analysis with log-rank tests and, when needed, pairwise comparison are shown in Table 4 and Figure 2 and Figure 3.

The Kaplan–Meier analysis of the patient groups according to the radicality of the surgery for primary OC (Figure 3A, Table 4) revealed a significant difference in the survival curves (χ^2^ = 22.61, *p* < 0.001). A higher slope characteristic for the curve determined for the group of patients that underwent non-radical surgery indicates a greater probability of death than for the group after radical surgery. The ES of the observed difference is positive and equal to 0.54, implying a higher hazard in the case of non-radical than in radical surgery.

Platinum resistance proved to be another crucial factor determining the survival time. Patients resistant to platinum were characterised by a significantly higher probability of event occurrence (*p* < 0.001). As in the previous case, the ES of the observed difference is positive and equal to 0.49, implying a higher hazard in a platinum-resistant group (Table 4, Figure 3B). The survival curves also differed significantly according to the ECOG score (χ^2^ = 20.86, *p* < 0.001), with a higher slope for patients assessed 1–2 compared to 0 scores (Figure 3C). The mean estimated survival time equals 64 ± 6.56 months for the 0 score and only 20.86 ± 3.25 months for 1 and 2 (Table 4). The negative ES indicates a higher probability of death in the group 1–2 than 0. Significant differences were further discovered for the AGO score, where the mean estimated survival time for patients assessed as positive was over three times higher than for patients who received a negative assessment (Figure 3D, Table 4). According to the survival analysis, the CCR index can be another valuable prognostic of the survival time (Table 4, Figure 3E). Patients who scored a CCR equal to 0 have an over two times longer estimated survival time (58.71 ± 6.23 months) compared to patients who received a CCR equal to 1 or higher (25.47 ± 4.09 months). The last variable for which significant differences in the mean survival times were revealed was the PCI, where for the scores lower than 15, the mean survival time is almost three times higher than for scores 15 and under (Table 4, Figure 3F).

**Figure 4:**
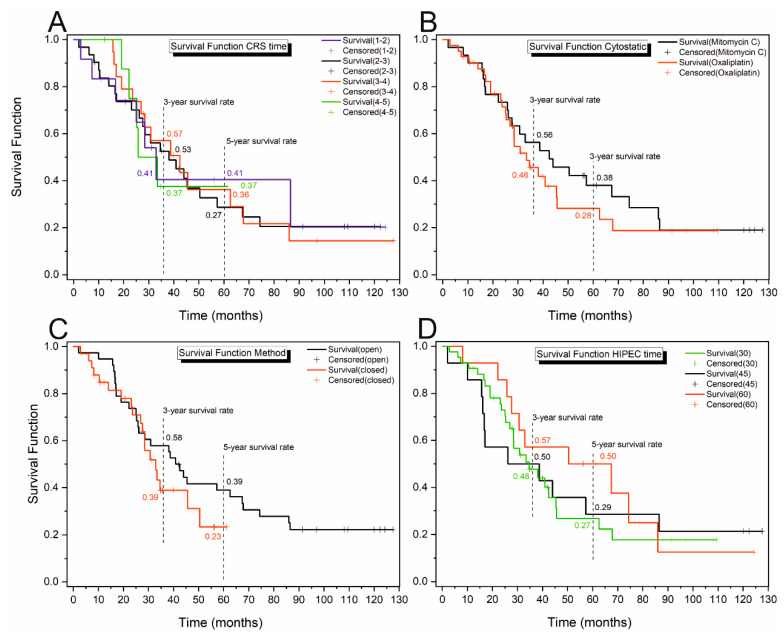
Survival curves for the CRS time (**A**), cytostatic (**B**), surgery method (**C**) and HIPEC time (**D**). Observations assigned a + were right-censored.

The differences between the groups were not statistically significant for the rest of the analysed variables (Table 5, Figure 4; however, in some cases, like the method, cytostatic or HIPEC time, it can be seen that the survival rates at specific times are quite different. Due to that, the survival probabilities were compared at 36 and 60 months for the mentioned variables using the log test and χ^2^ statistics (Table 5) [20]. The survival rates for the specific time points did not show significant differences for any variable over three years. However, a difference was observed for the HIPEC time over a 5-year period. The survival probability was found to be significantly higher for the procedure that lasted for 60 min compared to 30 or 45 min.

### 3.3. Cox Regression

Separate univariate Cox regressions were performed for each factor (Table 6) to determine the range of variables with a significant influence on the hazard function h(t).

**Figure 5:**
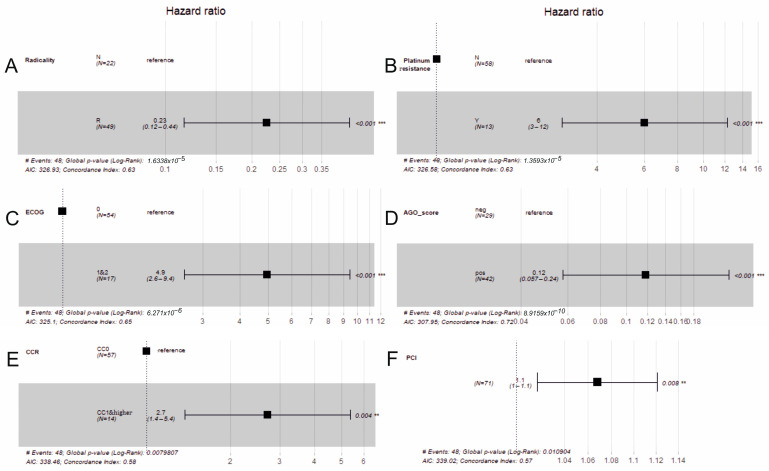
The forest plots of the hazard ratios (HRs) determined for the radicalness (**A**), Platinum resistance (**B**), ECOG (**C**), AGO score (**D**), CCR (**E**) and PCI (**F**) with the corresponding statistics: Akaike information criterion (AIC), concordance index (C-index) and *p*-value. **—*p* < 0.05, ***—*p* < 0.001.

Only the radicality, platinum resistance, ECOG, AGO score, CCR and PCI have statistically significant β coefficients among the tested variables. The coefficients determined for the radicality and AGO score have a negative sign (the radicality reference was N, and the AGO score reference was negative) and HR < 1, which means that there is a lower risk of event occurrence compared to the reference group (higher survival rates for radical surgery and positive AGO score). The platinum resistance (reference no), ECOG (reference was 0), CCR (reference CCR1 and higher) and PCI have positive coefficients and HR > 1, indicating that the survival probability is lower than for ECOG = 1, CCR1 and higher and in the case of higher PCI values. Figure 5 presents forest plots of the HR determined for significant variables with statistics assessing the model quality and fit (C-index, Akaike information criterion—AIC). It can be noticed that the higher value of the C-index was achieved by the model, including the AGO score (0.72), which simultaneously has the lowest AIC criterion (307.95). This means that the loss of information for that model is the lowest.

The multivariate Cox regression model, built in the next step, included only those variables that proved significant in the univariate analysis (Table 6). The *p*-value for the overall test (Wald test = 47.78) was lower than 0.001, indicating that the model was also significant. The C-index, a measure evaluating the predictive accuracy of a Cox proportional hazards model, was equal to 0.796 (±0.04), which means that, in 79.6% of the pairs of subjects where it is possible to make a comparison, the subject with the higher predicted risk (according to the Cox model) experiences the event (e.g., death) first. The model could be written as follows:

$$h\left(t\right)=h_{0}\left(t\right)exp(0.552\cdot Radicality+1.274\cdot Platinum\;resistance-0.237\cdot \;ECOG-2.858\cdot AGO\;score-0.592\cdot CCR+0.045\cdot PCI)$$

where t—survival time, h(t)—hazard function determined by covariates (x1, x2, …, xn), and h0(t)—baseline hazard.

Based on the results (Table 7), only the covariates platinum resistance and AGO score remained significant (*p* < 0.05). The *p*-value for the AGO score was 0.001, with a hazard ratio HR = 0.057, indicating a strong relationship between the patients’ AGO score and an increased risk of death if diagnosed negatively—the risk of event occurrence is only 5.7% for the group in question compared to the reference group, assuming other variables in the model are held constant. The HR for platinum resistance of 3.574 shows, in turn, that the risk of event occurrence is over 3.5 times higher for platinum-resistant patients. The AGO score and platinum resistance could then be used as excellent indicators of the survival probability.

**Figure 6:**
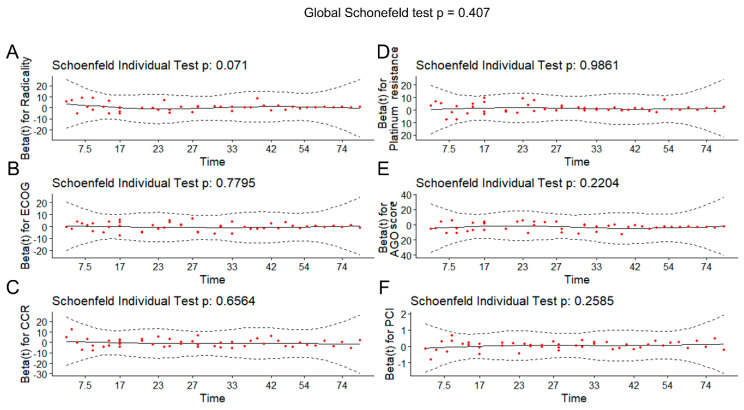
The scaled Schoenfeld residuals (red dots) against the transformed time for (**A**) radicality, (**B**) ECOG, (**C**) CCR, (**D**) platinum resistance, (**E**) AGO score and (**F**) PCI. The solid line is a smoothing spline fit to the plot, with the dashed lines representing a ±2-standard error band around the fit.

The model built was then assessed according to the assumption of proportional hazards (PHs) across different covariates. The results (Table 8, Figure 6) showed that the assumption of proportional hazards appears to be supported, and there is no pattern with time, as the relationship between the residuals and time proved to not be significant (*p* > 0.05).

**Figure 7:**
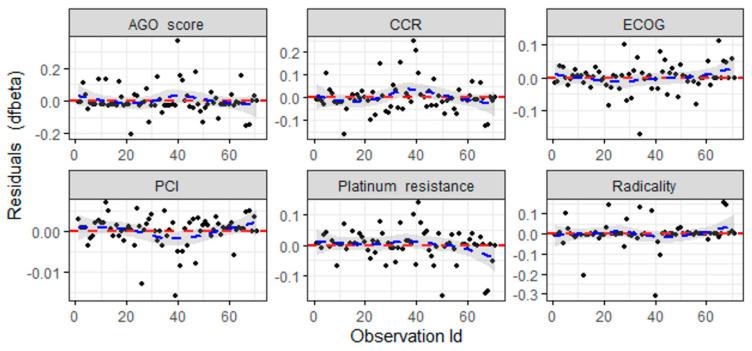
Index plots of the dfBETA (black dots) for the Cox regression of time to death on the AGO score, CCR, ECOG, PCI, platinum resistance and radicality. The dashed red horizontal line represents a value of 0 for dfBETA; the dashed blue line represents the locally weighted scatterplot smoothing (LOWESS) fit.

The index plots (Figure 7) show that the comparison of the magnitudes of the most significant dfBETA values to the regression coefficients suggests that none of the observations is individually influential, even though some of the dfBETA values for the AGO score and CCR and radicality are larger than the assumed threshold of 0.24. It could be assumed that all the necessary assumptions required for the Cox proportional hazards model were rigorously tested and satisfied. The above assessments collectively confirm that the Cox regression model is well-fitted to our data, providing reliable and robust estimates of the hazard functions associated with the studied covariates.

### 3.4. Principal Component Analysis

To discover the structure and regularities in the relationships between the variables, principal component analysis (PCA) was used to obtain a new space defined by new variables created based on the studied ones. The Bartlett statistics was determined before the analysis to test the hypothesis H0 that all the correlation coefficients are equal against H1 that not all the correlation coefficients are equal. The χ^2^ statistics = 348 and *p*-value < 0.001 indicate that the H0 hypothesis should be rejected. Additionally, the KMO (Kaiser–Mayer–Olkin) coefficients (Table 9) are relatively high for all the variables, excluding the method and platinum-resistant variables. This shows a strong relationship between the input variables (apart from method and platinum resistance) and confirms the reasonableness of using principal component analysis.

**Figure 8:**
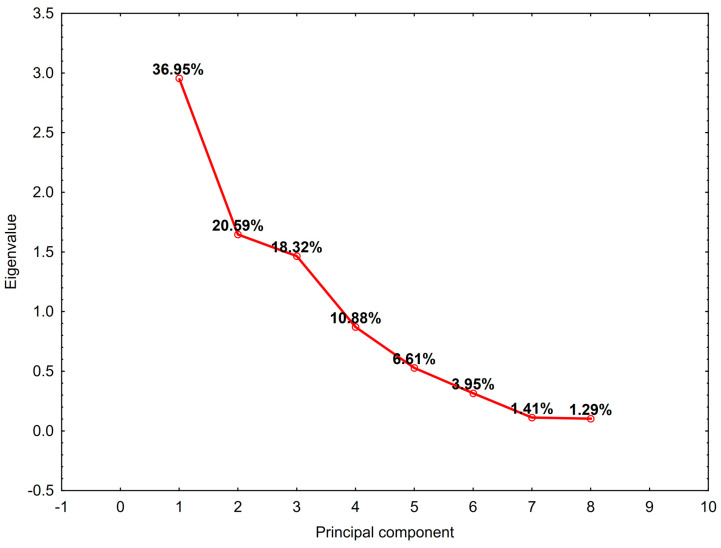
The scree plot displaying the eigenvalues (the amount of variance in the original data) captured by each principal component.

For the analysis, uncorrelated data were chosen and standardized. Active cases were those marked as uncensored. The selection of the ideal number of variables was determined using scree plot (Figure 8) and the Kaiser–Guttman criterion, indicating that principal components with eigenvalues exceeding one and accounting for a minimum of 75% of the overall variability are significant. In this manner, the principal components were reduced to three (PC1, PC2, and PC3), with 76% of the overall variance explained.

**Figure 9:**
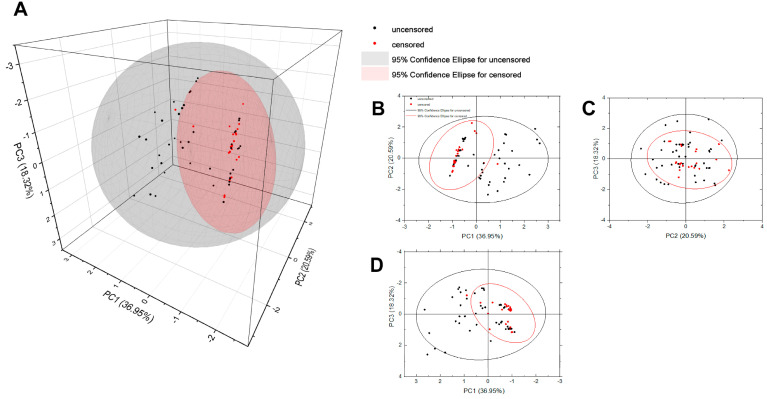
The projection of active (uncensored, black dots) and additional (censored, red dots) cases with 95% confidence ellipses in a new space created by (**A**) three components PC1, PC2 and PC3, (**B**) components 1 (PC1) and 2 (PC2), (**C**) components 2 (PC2) and 3 (PC3), and (**D**) components 1 (PC1) and 3 (PC3).

As can be seen in Table 10, the first component (PC1) is mainly built with variables contributing to the patient’s condition before the CRS and HIPEC procedure. The second component (PC2) shows the quality of the surgery, while the third (PC3) represents the HIPEC characteristics. As shown in Figure 9A, the chosen components create a new space in which the analyzed cases could be projected.

It is clearly visible (Figure 9B,D) that additional cases (censored) representing patients for whom the event did not occur during the observation period are mainly located on the negative side of the PC1 component. Considering that PC1 is primarily built with the negatively correlated radicality and AGO score and the positively correlated ECOG, it might be concluded that patients who underwent radical primary surgery received positive AGO scores and lower values of ECOG and had a higher possibility to live longer. The second component (PC2), which includes the positively correlated CRS time, CCR and PCI, divides the cases into groups on the positive and negative sides of the axis (Figure 9B,C). However, a few more of the additional samples are on the negative side, indicating that patients with lower PCI and CCR indices and shorter CRS times have a higher probability of survival. The role of PC3 is challenging to assess. Nevertheless, given that censored cases are positioned at or near the negative side of PC3 (as shown in Figure 9C,D), it can be hypothesized that the factor associated with surgery characteristics, described by a negatively correlated HIPEC time and a positive correlation with Cytostatic, indicates a direct proportional relationship between the HIPEC time and survival, and an inverse relationship between Cytostatic (where 1 represents Oxaliplatin and 0 represents Mitomycin C) and survival. This suggests that a longer HIPEC time is associated with better survival outcomes, whereas using different cytostatic agents has varying impacts on survival, with Oxaliplatin offering a slightly lower chance of survival than Mitomycin C.

## 4. Discussion

The findings of the current study contribute to the growing body of literature exploring the prognostic factors in ROC. This study has shown that the radicality of surgery for primary OC, the performance status as assessed by the ECOG, AGO score, and the PCI significantly impact the OS of patients undergoing CRS with HIPEC for ROC. These factors have been independently correlated with OS and merit comparison with the findings from other research.

The expected survival period after the recurrence of OC is roughly 12 to 18 months. Less than 10% of patients survive for more than 5 years after undergoing standard salvage CTH treatment [21]. The current study group’s Kaplan–Meier survival analysis demonstrates a mean survival of 52.7 months post-treatment with CRS and HIPEC, accompanied by 3-year and 5-year survival rates of 0.50 and 0.33, respectively.

In comparison, other studies have reported varying survival rates for similar patient cohorts treated with CRS and HIPEC. In the academic discourse, the application of HIPEC in the context of ROC is nuanced and based on various clinical studies.

The prospective randomized phase III trial by Spiliotis et al. plays a pivotal role in endorsing HIPEC, demonstrating its effectiveness in treating ROC. This study meticulously evaluated the efficacy of HIPEC when applied at the first recurrence, inclusive of both platinum-sensitive and platinum-resistant patients. The assignment of 60 participants into two distinct groups—one receiving CRS coupled with systemic CTH, and the other receiving an augmented protocol of CRS with HIPEC, followed by systemic CTH—allows for a comparative analysis. Remarkably, the results indicated a mean OS of 26.7 months for the HIPEC group, which is notably higher than the 13.4 months observed in the control group. This disparity was statistically significant (*p* < 0.01), with both the platinum-sensitive and platinum-resistant subgroups within the HIPEC cohort exhibiting a similar OS of 26.8 and 26.6 months, respectively. In contrast, significant differences were noted within the control arm—15.2 months for the platinum-sensitive subgroup versus 10.2 months for the platinum-resistant subgroup (*p* < 0.01) [22]. In their research, Spiliotis et al. found a mean overall survival (OS) of 26.7 months in the HIPEC group. Despite being lower than our own results, this outcome still denotes a significant advancement over the control group’s mean OS of 13.4 months.

In contrast, the study conducted by Zivanovic et al. presents a different perspective on the effectiveness of HIPEC, specifically when carboplatin is used in secondary CRS for platinum-sensitive ROC. In this phase II RCT, the utility of carboplatin-based HIPEC followed by five cycles of CTH was juxtaposed against CRS alone, followed by a similar CTH regimen. Diverging from Spiliotis et al., Zivanovic et al. did not observe a survival advantage with the HIPEC addition, with the analysis indicating a statistically significant 18% increased risk of disease progression or death for patients undergoing HIPEC [23]. This starkly contrasts with our results and those of Spiliotis et al., potentially due to the use of carboplatin, which may have a differential efficacy in HIPEC compared to the agents used in our study, such as Mitomycin C or Oxaliplatin.

Further insights were provided by the CHIPOR phase III RCT presented in June 2023 at the ASCO meeting in Chicago. This extensive, decade-spanning multicentric trial across 31 institutions focused on patients experiencing their initial platinum-sensitive ROC. Patients underwent six cycles of platinum- and taxane-based CTH, with or without bevacizumab. Patients eligible for complete CRS post-CTH were randomized to either receive HIPEC or not. The findings were compelling, revealing a notable enhancement in both OS (54.3 vs. 45.8 months) and peritoneal PFS (13.1 vs. 12.2 months). Additionally, an improvement in global PFS was also noted (10.2 vs. 9.8 months) [24].

The collective evidence from these studies is reflected in the current clinical guidelines, which, as of the latest consensus, do not uniformly endorse HIPEC in conjunction with primary or secondary CRS. However, opinions on the integration of HIPEC with interval CRS range from supportive to not recommended among various global societies and organizations [13,25].

The discourse around the application of HIPEC in ROC management remains dynamic, with ongoing trials and updates to clinical guidelines expected to further refine our understanding of its role in improving patient outcomes. 

The prognostic impact of the extent of surgery for primary OC is a subject of clinical significance. A compelling correlation has been observed, where patients who underwent comprehensive CRS, achieving complete resection of cancerous tissues during the initial surgical intervention, have a notably extended mean survival time [26]. In our study, survival in patients who underwent radical surgery for primary OC approximates an average of 65 months, which is markedly superior compared to those who had non-radical surgeries, where the mean survival time was 24.5 months (*p* < 0.001).

It is suggested that patients who not only had a complete tumor resection initially but also those who experienced a disease-free interval surpassing 12 months post-primary operation might derive the most substantial benefit from subsequent surgical intervention upon disease recurrence. Offering radical surgery to this subset of patients could significantly augment the effectiveness of second-line CTH regimens. It is noteworthy that the efficacy of CTH alone may be suboptimal in the context of bulky recurrent disease, emphasizing the importance of surgical intervention in this patient population [26].

Multivariate Cox regression analysis conducted by Jänicke et al. identified the presence of a residual tumor following the second surgical intervention as the independent predictor of survival, with a relative risk of 4.7 [26]. The extent of surgery for recurring cancers is a critical factor that impacts patient prognosis. Therefore, improving the effectiveness of surgical interventions is essential for better prognostic outcomes. In this context, the integration of HIPEC following CRS is emerging as a promising approach to augment local control of the disease. The strategy of using HIPEC post-CRS is grounded in the philosophy of achieving maximal control over the disease process by utilizing the synergistic effects of hyperthermia and localized high-concentration chemotherapy to enhance the destruction of cancer cells while preserving surrounding healthy tissues.

The ECOG performance status is a well-documented prognostic factor across oncological conditions, including OC. Patients with an ECOG score of “0” exhibited a mean survival time of 64 months, indicating better outcomes for patients with full functionality. In contrast, those with ECOG scores of “1–2” had a considerably lower mean survival time of 20.9 months. The concurrence between higher ECOG scores and reduced survival rates is in accordance with the research conducted by Asp et al., which further supports the notion that patients with superior performance statuses typically achieve more favorable outcomes [27].

Upon careful analysis of the implications of platinum resistance in ROC, it is evident, through a comprehensive review of the literature, that platinum resistance plays a substantial role in compromising both survival and therapeutic effectiveness, a finding that is supported by the results of our study. Davis et al. delineate the challenging prognosis associated with platinum-resistant OC, notably characterised by diminished CTH response rates and a median survival under 12 months. This highlights the importance of finding new treatments and better ways to measure patient progress in this challenging group of patients [28]. Moreover, the research conducted by Pinato et al. indicates that the utilization of dose-dense CTH regimens could potentially augment the effectiveness of treatment in cases displaying resistance to platinum-based therapies. This approach presents a prospective strategy to address clinical resistance and enhance patient response rates [29]. Furthermore, a study conducted in Poland by Bodnar et al. utilizing real-world clinical data demonstrates that second-line platinum-based treatments can substantially enhance survival rates for individuals with a platinum-free interval (PFI) of 3–6 months, thereby highlighting the influential role of treatment timing and resistance dynamics in therapeutic efficacy [30]. The research conducted by Marchetti et al. emphasizes the crucial importance of identifying robust biomarkers and therapeutic targets to comprehend the molecular mechanisms driving platinum resistance. The importance of these advancements cannot be overstated, as they contribute to the optimization of treatment regimens and the enhancement of prognostic assessments in ROC [31]. Collectively, these studies elucidate the complex nature of managing platinum-resistant ROC and highlight the necessity for targeted research and innovative therapeutic strategies to address this formidable challenge.

A positive AGO score is linked to a higher mean survival time of 74.32 months, showing its importance as a prognostic factor. In the multivariate Cox regression analysis, the AGO score was a significant variable, proving its validity as a strong predictor of survival outcomes among various factors. Patients with a negative AGO score have a higher risk of mortality compared to those with a positive score, emphasizing its critical prognostic value.

Harther et al. confirmed that the AGO score accurately predicts surgical outcomes. They found that patients with a positive score had a median OS of 63.9 months after complete resection, compared to 48.4 months for those with a negative score. This difference was not statistically significant in the multivariate analysis [32]. Another study by Muallem et al. in 2015 supported these findings. They showed that the AGO score can predict operability during secondary cytoreductive surgery. Even patients with negative scores still have a significant chance of achieving optimal tumor resection [33].

Armbrust et al. conducted a meta-analysis that included health-related quality of life measures and clinical factors, such as the AGO score, to predict short-term mortality. They developed a risk score, which showed that a low AGO score was associated with a high risk of short-term mortality [34]. Another study by van de Laar et al. found that although the AGO score had a high positive predictive value for complete secondary cytoreduction, it also had a high false-negative rate. This means that some patients who could benefit from surgery might be overlooked [35].

The AGO score’s relevance extends to patients undergoing HIPEC. Bogani et al. found that the AGO score could identify patients likely to achieve CCR0 in secondary CRS for ROC. However, the study also reported that a considerable proportion of patients who did not meet the AGO criteria still achieved complete cytoreduction, suggesting the need for broader evaluative measures [36].

These studies corroborate the AGO score as a valuable tool for predicting surgical outcomes and survival in ROC, aligning with the findings of our investigation. While the AGO score is indeed informative, its application should be nuanced, considering the potential for underestimating the candidacy of patients for beneficial surgery. Future research ought to refine these prognostic models, potentially integrating them with other predictive markers to enhance the precision of patient selection for secondary cytoreductive surgery and HIPEC.

Furthermore, the PCI emerged as a noteworthy indicator of survival. Generally, patients with lower PCI scores, indicating less widespread disease, exhibited higher rates of survival. This underscores the significance of the disease extent when it comes to prognosis and treatment strategies. In the context of surgical management of ROC, attaining complete cytoreduction, which often aligns with lower PCI scores, becomes crucial for a favorable prognosis. Our study revealed that patients with a PCI exceeding 15 experience a substantial decline in survival rates.

The PCI is widely recognized as a crucial prognostic indicator in advanced OC. It plays a vital role in guiding the extent of the surgical intervention and predicting patient outcomes, and its importance cannot be underestimated. Tentes et al. conducted a study that demonstrated a significant relationship between the PCI and survival, underscoring the value of a thorough assessment of peritoneal spread in advanced ovarian cancer cases [37]. Similarly, Chéreau et al. found a strong correlation between various carcinomatosis scores and the PCI in predicting resectability and survival outcomes. This highlights the necessity for alternative ranking systems beyond FIGO staging to inform surgical decisions and prognosis [38]. Elzarkaa et al. further supported these findings by reporting that a higher PCI score is associated with worse survival, thus indicating its utility in predicting the success of complete surgical cytoreduction [39].

The prognostic value of the PCI was also demonstrated by Gasimli et al. They found that higher PCI scores were associated with poorer OS and PFS, particularly in patients with primary advanced OC. They determined the optimal cut-off value to be 15. Multivariate analysis revealed that age, residual tumor, and PCI were independent prognostic factors for survival. Patients with advanced OC who had a PCI greater than 10 showed a significant correlation with a poor prognosis [40].

Fagan et al. suggested that a PCI score of ≤20 was associated with a high likelihood of complete cytoreduction in advanced OC [41]. This emphasizes the importance of the PCI as an independent prognostic indicator in preoperative assessments. It is crucial to critically evaluate the appropriateness of CRS in cases with elevated PCI levels, considering the potential for serious complications. This evaluation ensures that the therapeutic benefits of invasive interventions outweigh the risks of postoperative complications. Thus, high PCI values may challenge the clinical justification for CRS, leading to consideration of alternative treatment strategies to optimize patient outcomes and minimize harm.

Lomnytska et al. identified the PCI as an independent predictor of high-grade complications after ovarian cancer surgery, highlighting its clinical relevance in perioperative planning and patient counseling [42]. Furthermore, Jónsdóttir et al. found the PCI to be an excellent predictor of operability and survival outcomes, with higher scores correlating with increased rates of complications and reduced chances of survival [43].

These studies provide strong evidence of the prognostic significance of the PCI in advanced OC patients. They emphasize its utility in surgical decision-making and predicting patient outcomes. By using the PCI, clinicians can stratify patients more accurately and tailor treatment strategies accordingly, potentially improving survival rates and quality of life.

The PCA was instrumental in elucidating the intricate interrelationships among various factors and their impact on patient outcomes post-treatment with CRS and HIPEC. The PCA effectively distilled the complexity of the data into three principal components, each highlighting different dimensions of the study’s variables.

A notable aspect of the PCA was the revelation of the correlation patterns. Positive correlations emerged between factors such as the radicality of surgery for primary OC and positive AGO scores, which were associated with longer survival. Conversely, higher ECOG scores, indicative of a lower level of patient functionality, correlated with poorer survival outcomes. The PCA also highlighted the role of the PCI in survival, with lower scores correlating with better outcomes.

Graphical representations from the PCA provided a visual digest of these correlations, demonstrating clear distinctions between patients with favorable and less favorable scores in terms of the radicality, AGO, and ECOG. This visualization accentuated the multifaceted nature of the factors influencing survival rates.

From a clinical perspective, these insights from the PCA are invaluable. They emphasize the need for a comprehensive approach in treatment planning, considering surgical aggressiveness, patient health, and tumor characteristics. This comprehensive view assists clinicians in tailoring treatment strategies for each patient, potentially enhancing outcomes.

The PCA analysis not only validated the importance of individual factors like the radicality of surgery, AGO score, and ECOG status but also provided a holistic view of their combined impact on survival in patients undergoing CRS and HIPEC. This analysis adds depth to our understanding of the complex dynamics involved in treating ROC.

Despite yielding promising results, this study has several limitations. The retrospective observational design employed in the study hinders the establishment of a clear cause-and-effect relationship between treatment methods and patient outcomes. This design is susceptible to selection bias and other variables that are not typically present in prospective trials. Moreover, the sample size is relatively small, comprising only 71 patients, which may not provide sufficient statistical power to detect smaller effect sizes or allow for generalization of the findings to a broader population of patients with ROC. Additionally, since the study was conducted at a single institution, the results may not be applicable to other settings due to potential variations in patient demographics, surgical expertise, and procedural specifics. The absence of a contemporaneous control group that did not undergo HIPEC treatment further limits the ability to directly attribute any improvements in survival solely to the HIPEC procedure. Furthermore, the study’s classification of platinum sensitivity may not fully capture the complexity of the tumor biology in ROC, which could potentially affect the accuracy of the study’s findings regarding the treatment outcomes.

## 5. Conclusions

The results of this study highlight several important factors that are associated with improved survival in patients with recurrent ovarian cancer undergoing HIPEC. These factors include radical surgery, better performance status, platinum sensitivity, positive AGO score, and lower PCI. Interestingly, platinum resistance and the AGO score were found to be strong predictors of patient outcomes. Therefore, it is crucial to take these factors into consideration when planning treatment and predicting prognosis for patients with ROC. Additionally, efforts to reduce the PCI through surgical intervention and other treatments are of the utmost importance in the strategy to enhance the survival outcomes in ovarian cancer. The PCA analysis highlights the multifactorial influences on ROC patients in relation to HIPEC and CRS, suggesting that a variety of patient-specific and treatment-related factors play a role in the outcomes. While these findings provide valuable insights, it is important to recognize that the evidence from this study is preliminary and not yet sufficient to draw definitive conclusions about the efficacy of HIPEC and CRS in ROC management. Therefore, our results should be considered as a foundation for further research rather than conclusive evidence. Future prospective studies with larger sample sizes will be crucial to validate these initial observations and enhance clinical decision-making.

## Figures and Tables

**Table 1:** Characteristics of the continuous variables.

Characteristics	Median	Q1	Q3
Survival time (months)	31.00	19.10	56.00
CRS time (minutes)	150.00	120.00	210.00
HIPEC time (minutes)	30.00	30.00	45.00
PCI	5.00	4.00	11.00

**Table 2:** Characteristics of the categorical variables along with statistical analysis of the structure indices.

Characteristic		Number of Patients n	Percentage
Radicality	N	22	31%
	R	49	69%
Platinum resistance	Y	13	18%
	N	58	82%
ECOG	0	54	76%
	1 and 2	17	24%
AGO score	Positive	42	59%
	Negative	29	41%
CCR	0	57	80%
	1 and higher	14	20%
Cytostatic	Mitomycin C	30	42%
	Oxaliplatin	41	58%
HIPEC method	Open	38	54%
	Closed	33	46%

**Table 3:** Quartile estimates of the overall survival function for women diagnosed with ovarian cancer after HIPEC therapy.

Percent Failures	Estimate	95% L.C.I.	95% U.C.I.
25	23.10	16.67	27.60
50	38.10	28.37	45.57
75	74.37	45.57	86.53

L.C.I.—lower bound of the confidence interval; U.C.I.—upper bound of the confidence interval.

**Table 4:** Mean estimated survival times with the SE and 95% CI and the quartile estimates, accompanied by the statistics, *p*-values and effect sizes (ES).

Variable		Mean Estimated Survival Time ± SE (CI) (Months)	Quartile Estimates (CI)			Statistics and *p*-Value
			25%	50%	75%	
Radicality	N	24.50 ± 2.23 (20.13; 28.87)	17 (14; 25)	25 (17; 28)	28 (27; 42)	χ^2^ = 22.61 *p* < 0.001 ES = 0.54 (large)
	R	65.33 ± 6.98 (51.64; 79.02)	26 (22; 41)	57 (39; 86)	-	
Platinum resistance	Y	17.87 ± 3.71 (10.59; 25.57)	10 (3; 17)	17 (10; 23)	24 (16; 25)	χ^2^ = 30.61 *p* < 0.001 ES = 0.49 (large)
	N	60.29 ± 6.11 (48.30; 72.27)	27.6 (22; 33)	44 (33; 67)	87 (63; 86)	
ECOG	0	64.00 ± 6.56 (51.14; 76.86)	28 (22; 38)	50 (35; 74)	-	χ^2^ = 20.86 *p* < 0.001 ES = −0.55 (large)
	1 and 2	20.86 ± 3.25 (14.49; 27.23)	10 (3; 17)	17 (10; 27)	27 (17; 42)	
AGO score	Positive	74.32 ± 7.33 (59.95; 88.86)	38 (26; 57)	67 (45; 87)	-	χ^2^ = 44.40 *p* < 0.001 ES = 0.86 (large)
	Negative	22.09 ± 2.18 (17.81; 26.37)	16 (7; 19)	24 (17; 27)	28 (25; 33)	
CCR	0	58.71 ± 6.23 (46.50; 70.91)	25 (17; 31)	44 (30; 67)	87 (63; 87)	χ^2^ = 8.609 *p* = 0.004 ES = 0.29 (medium)
	1 and higher	25.47 ± 4.09 (17.44; 33.50)	16 (3; 27)	25 (16; 33)	33 (25; 42)	
CRS time (h)	1–2	54.92 ± 14.82 (25.87; 83.96)	17 (7; 33)	33 (17; 87)	87 (28; 87)	χ^2^ = 0.017 *p* = 0.999 ES = not relevant
	2–3	50.69 ± 7.73 (35.55; 65.84)	17 (10; 30)	38 (26; 57)	67 (44; 74)	
	3–4	52.55 ± 9.15 (34.61; 70.50)	24 (17; 39)	42 (27; 68)	68 (42; 86)	
	4–5	38.67 ± 6.32 (26.27; 51.06)	22 (19; 33)	26 (22; 33)	-	
Cytostatic	Mitomycin C	56.24 ± 7.91 (40.74; 71.74)	22 (16; 33)	42 (26; 74)	86 (50; 87)	χ^2^ = 0.349 *p* = 0.554 ES = −0.09 (small)
	Oxaliplatin	45.88 ± 6.25 (33.63; 58.13)	23 (17; 28)	33 (27; 46)	63 (41; 67)	
Method	Open	57.30 ± 6.98 (43.62; 70.98)	22 (17; 31)	41 (26; 67)	86 (57; 87)	χ^2^ = 1.204 *p* = 0.272 ES = −0.11 (small)
	Closed	35.35 ± 3,59 (28.31; 42.40)	23 (10; 28)	33 (28; 50)	50 (33; 50)	
HIPEC time (min)	30	45.58 ± 6.03 (33.77; 57.40)	24 (17; 28)	35 (28; 45)	62 (41; 68)	χ^2^ = 0.603 *p* = 0.740 ES_30–60_ = 0.09 (small) ES_rest_ = not relevant
	45	50.91 ± 12.08 (27.25; 74.58)	16 (10; 39)	39 (16; 86)	86 (26; 86)	
	60	58.12 ± 9.95 (38.62; 77.63)	28 (22; 67)	67 (28; 86)	86 (50; 86)	
PCI	0–4 ^a^	63.14 ± 10.23 (43.10; 83.19)	25 (17; 45)	45 (28; 65)	-	χ^2^ = 9.403 *p* = 0.024 ES_0–4–15&under_ = −0.10 (small) ES_rest_ = not relevant
	5–9 ^a^	50.21 ± 9.89 (30.83; 69.58)	17 (10; 28)	29 (17; 44)	74 (31; 74)	
	10–14 ^a^	53.54 ± 6.92 (39.98; 67.10)	31 (23; 50)	50 (35; 67)	68 (50; 86)	
	15 and above ^b^	21.67 ± 3.19 (15.42; 27.93)	16 (2; 25)	19 (16; 26)	27 (19; 33)	

SE—standard error, CI—confidence interval, ES—effect size, a and b indices show differences among groups.

**Table 5:** Comparison of the 3- and 5-year survival probabilities with the 95% confidence interval and statistical analysis results.

Group		3-Year Survival Rate (CI)	5-Year Survival Rate (CI)
Method	Open	0.579 (0.557; 0.600)	0.390 (0.358; 0.422)
	Closed	0.395 (0.349; 0.441)	0.237 (0.154; 0.330)
Statistics and *p*-value		χ^2^ = 1.866 *p* = 0.172	χ^2^ = 1.061 *p* = 0.303
Cytostatic	Mitomycin C	0.563 (0.533; 0.591)	0.380 (0.337; 0.423)
	Oxaliplatin	0.458 (0.428; 0.487)	0.283 (0.233; 0.336)
Statistics and *p*-value		χ^2^ = 0.716 *p* = 0.397	χ^2^ = 0.566 *p* = 0.452
HIPEC time (min)	30–45	0.483 (0.463; 0.503)	0.274 (0.240; 0.308)
	60	0.587 (0.528; 0.641)	0.513 (0.444; 0.578)
Statistics and *p*-value		χ^2^ = 0.539 *p* = 0.463	χ^2^ = 3.034 *p* = 0.041

CI—confidence interval.

**Table 6:** Univariate Cox regression analysis results.

Variable	Reference Level	β	HR	(95% CI for HR)	Wald Test	*p*-Value
Radicality	Non-radical	−1.50	0.23	(0.12; 0.44)	19.00	<0.001
Platinum resistance	No	1.79	6.01	(2.95; 12.2)	18.93	<0.001
ECOG	0	1.60	4.90	(2.6; 9.4)	23.00	<0.001
AGO_score	Negative	−2.10	0.12	(0.057; 0.24)	34.00	<0.001
CCR	1 and higher	0.99	2.71	(1.37; 5.37)	7.04	0.004
CRS_time	-	0.01	1.00	(1.00; 1.01)	0.15	0.879
Cytostatic	Mitomycin C	0.18	1.20	(0.67; 2.1)	0.37	0.550
Method	Closed	−0.34	0.71	(0.38; 1.3)	1.20	0.280
HIPEC_time	-	−0.01	0.99	(0.97; 1)	0.76	0.380
PCI	-	0.07	1.10	(1; 1.1)	7.10	0.008

**Table 7:** Multivariate Cox proportional hazard regression results (β—regression coefficient, HR—hazard ratio, -HR—hazard ratio in respect of the opposite group or direction, z—statistics), with the radicality, platinum resistance, ECOG, AGO score, CCR and PCI as covariates.

Variable	Reference	β ± SE	HR (CI)	-HR	z	*p*-Value
Radicality	Non-radical	0.552 ± 0.095	1.738 (0.746; 5.942)	0.576	1.076	0.282
Platinum resistance	No	1.274 ± 0.045	3.574 (1.476; 8.635)	0.279	2.823	0.005
ECOG	0	−0.237 ± 0.036	0.788 (0.311; 2.000)	1.269	−0.500	0.617
AGO score	Negative	−2.858 ± 0.027	0.057 (0.013; 0.249)	17.418	−3.815	0.001
CCR	1 and higher	−0.592 ± 0.043	0.553 (0.191; 1.604)	1.809	−1.090	0.276
PCI	-	0.045 ± 0.015	1.047 (0.987; 1.121)	0.964	1.314	0.189

CI—confidence interval, SE—standard error.

**Table 8:** Proportional hazard assumption—Schoenfeld test results.

Variable	χ^2^	df	*p*-Value
Radicality	3.259692	1	0.071
Platinum resistance	0.000302	1	0.986
ECOG	0.078403	1	0.779
AGO score	1.501777	1	0.220
CCR	0.197870	1	0.656
PCI	1.277033	1	0.258
GLOBAL	6.146453	6	0.407

**Table 9:** KMO criterion for the PCA analysis.

General KMO	Radicality	ECOG	AGO Score	CRS Time	CCR	Cytostatic	HIPEC Time	PCI	Method	Platinum Resistance
0.492	0.455	0.490	0.540	0.581	0.653	0.423	0.445	0.567	0.073	0.038

**Table 10:** Factor loadings.

Variable	PC1	PC2	PC3
Radicality	−0.633	0.154	0.350
ECOG	0.565	−0.019	−0.463
AGO score	−0.816	0.106	0.479
CRS time	0.316	0.561	0.448
CCR	0.368	0.709	−0.082
Cytostatic	0.529	−0.548	0.605
HIPEC time	−0.458	0.508	−0.591
PCI	0.504	0.693	0.292

The green indicates variables that have the most decisive influence on the particular component.

## Data Availability

No new data were created or analysed in this study. Data sharing is not applicable to this article.
